# Cerebral Venous Thrombosis Revealing Primary Sjögren Syndrome: Report of 2 Cases

**DOI:** 10.1155/2013/747431

**Published:** 2013-01-03

**Authors:** A. Mercurio, M. Altieri, V. M. Saraceni, T. Paolucci, G. L. Lenzi

**Affiliations:** ^1^Department of Neurological and Psychiatric Sciences, Sapienza University of Rome, Viale dell'Università 30, 00185 Rome, Italy; ^2^Department of Physical Medicine and Rehabilitation, Sapienza University of Rome, P.le Aldo Moro, 5, 00185 Rome, Italy

## Abstract

Sjögren syndrome (SS) is an autoimmune disease of the exocrine glands, characterized by focal lymphocytic infiltration and destruction of these glands. Neurologic complications are quite common, mainly involving the peripheral nervous system (PNS). The most common central nervous system (CNS) manifestations are myelopathy and microcirculation vasculitis. However, specific diagnostic criteria for CNS SS are still lacking. We report two cases of primary SS in which the revealing symptom was cerebral venous thrombosis (CVT) in the absence of genetic or acquired thrombophilias.

## 1. Introduction

The prevalence of neurological involvement in Sjögren's syndrome (SS) ranges between 8% and 70% [1, 2]. The central nervous system (CNS) is involved in 1.5%–36% of SS patients, but its prevalence might be underestimated [2]. Indeed, CNS manifestations in SS are various and may involve the entire neuroaxis. This variability of neurologic presentation may delay the diagnosis [3].

Headache is the main CNS complication in SS. In addition, subclinical tissue injury may be determined by magnetic resonance imaging (MRI). The diagnosis of systemic disease is still based on clinical features and laboratory findings but some characteristic MRI findings exist [4]. Ferreiro et al. first reported a patient with diffuse angiographic changes, suggesting that CNS vasculitis may be the symptom of onset in some SS patients [5]. 

Antiphospholipid antibodies (aPL) are observed in 2%–37% of SS patients and may be responsible for thromboembolic events and fetal loss [6]. Nevertheless, thrombosis may also occur in the absence of aPL. To date, only one case of cerebral venous thrombosis (CVT) as the symptom of onset of SS has been reported. It was associated with myeloradiculopathy [7]. 

Herein, we present two cases of isolated transverse sinus (TS) thrombosis as the revealing symptom of primary SS. The patients had no genetic or acquired thrombophilias. 

## 2. Material and Methods 

### 2.1. Case  1

 A 43-year-old woman was admitted to our neurological ward because of severe headache associated with vomiting and generalized seizures. The pain was restricted to the left temporal area and irradiating to the neck. Her past clinical history was unremarkable except for oral and ocular sicca syndrome and recurrent cavities. 

**Figure 1:**
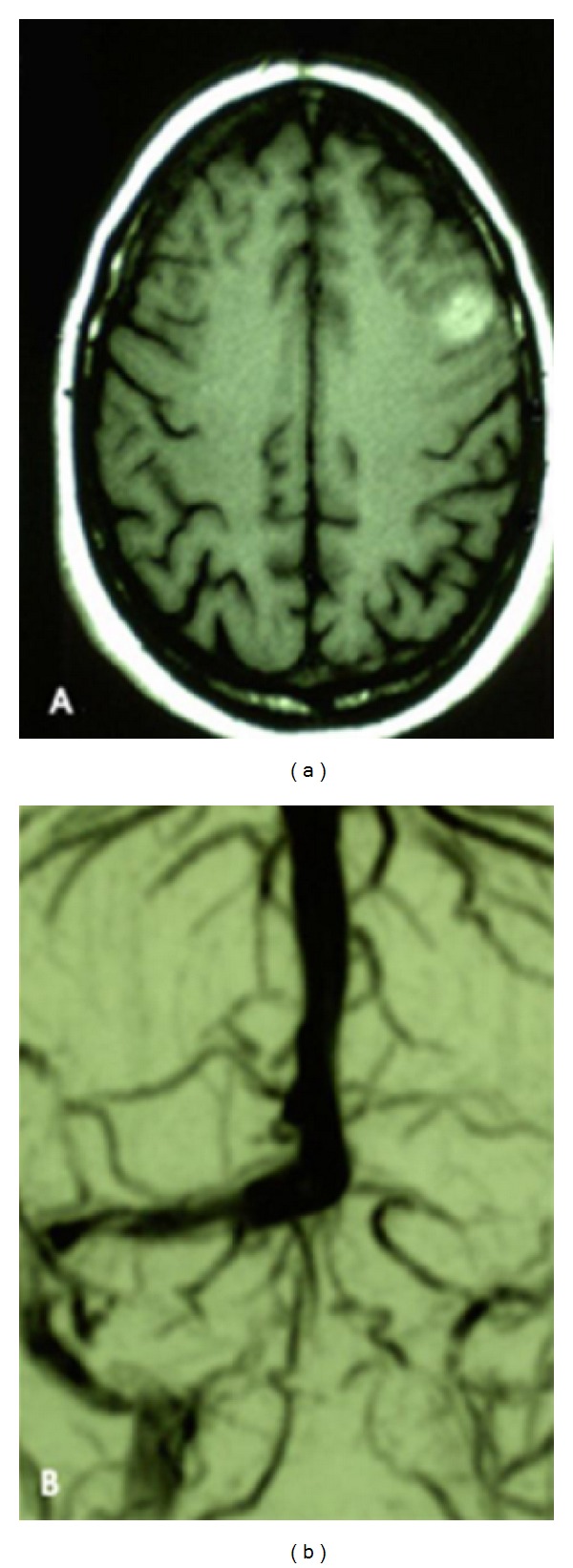
Patient 1: MRI revealed a left parietal hematoma, while the MR venography (MRV) showed an ipsilateral TS thrombosis.

Brain MRI revealed a left parietal hematoma, while the MR venography (MRV) showed an ipsilateral TS thrombosis (Figure 1). She was treated with IV heparin followed by oral anticoagulant. 

The patient underwent an extensive screening for coagulation disorder, included in the screening for thrombophilias, as we normally perform in any patient with CVT [8]. It includes protein C deficiency, protein S deficiency, antithrombin deficiency, activated protein C resistance, Leiden mutation of factor V gene, G20210A prothrombin mutation, homocysteine, and MTFHR mutations. The patient was also screened for the presence of autoantibodies: aPL, ANA, ENA, ANCA, and AMA. The aPL tested IgG and IgM anticardiolipin antibodies (aCL), IgG and IgM anti-*β*2 glycoprotein-1 antibodies (a*β*2GPI), and lupus anticoagulant (LA) according to the guidelines of the International Society on Thrombosis and Haemostasis [9]. 

The patient showed positive ANA (+++), ENA, and anti-Ro/SS-A antibodies. aPLA (repeated in other two occasions at least 12 weeks apart) were negative. Since the parotid scintigraphy, the biopsy of minor salivary glands, Schirmer test, and BUT were positive, the European criteria of Vitali for SS were satisfied (Table 1) [10]. She was treated with hydroxychloroquine and at the 3-year followup she had no clinical or radiological relapse and fully recovered. No delayed recanalization was observed at the MRV. 

### 2.2. Case  2

**Figure 2:**
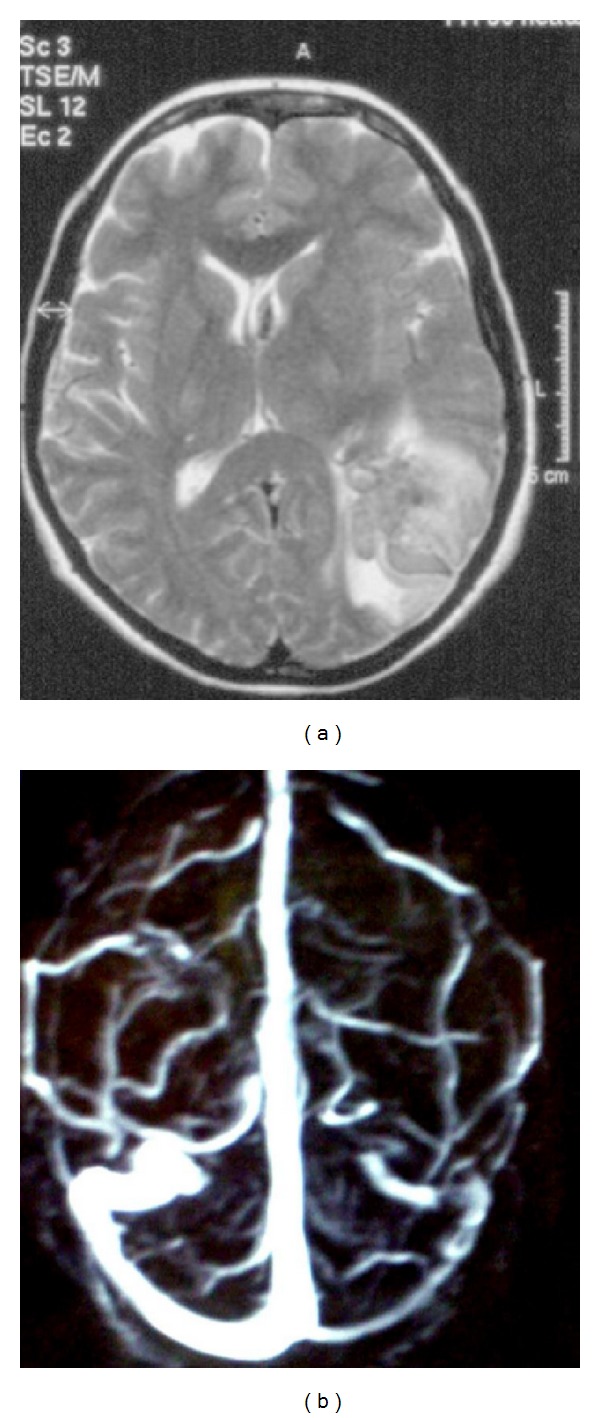
Patient 2: MRI showed a left temporal hematoma while the MRV revealed a left TS thrombosis.

 A 44-year-old woman admitted to our ward for the sudden onset of intracranial hypertension syndrome associated with right hemiparesis and ipsilateral seizures followed by secondary generalization. The patient had a clinical history of recurrent otitis, mucosal dryness, and recurrent hands' arthralgias. The brain MRI showed a left temporal hematoma while the MRV revealed a left TS thrombosis (Figures 2(a) and 2(b)). She was treated with LMWH followed by oral anticoagulant.

The same screening for coagulation disorder described previously was applied, and the patient showed positivity to ANA (+++), pANCA, antiRo/SS-A, and antiLa/SS-B antibodies. Also RA test was positive. aPL were negative. The diagnostic workup allowed the diagnosis of primary SS (Table 1). Hydroxychloroquine was introduced and at the 5-year followup she had no clinical or radiological relapse and fully recovered. Again, at the MRV no delayed recanalization was observed.

## 3. Discussion

 SS is an autoimmune disorder, more prevalent in young women (0.5%–1% of the general population with a F/M ratio = 9 : 1), HLA DR3-related and characterized by a lymphocyitic aggression of the exocrine glands [1]. Extraglandular manifestations are common. CNS involvement may be represented by myelitis, vasculitis of the microcirculation, associated with white matter lesions, aseptic meningitis, and encephalopathy [2, 11]. Arterial stroke has also been reported [2, 4, 12]. 

In the sole other case of CVT in SS patient previously described [7], the revealing symptom of SS was a myelitis, followed by TS thrombosis. Also in that case, no thrombophilic factors were detected. 

According to Hughes and Khamashta there might be 3 different possibilities to explain thromboembolic events occurring in seronegative patients: (1) a seronegative phase of a previously positive aPL; (2) conventional laboratory testing failing to pick up cases with antibodies directed against different phospholipids or proteins cofactors; (3) the presence of a different coagulopathy [13]. The diagnosis of seronegative aPL has been suggested for patients with clinical manifestations indicative of aPL but with persistently negative results in the commonly used assays to detect aCL antibodies, a*β*2GPI, and LA. To date the best management of these patients is still unclear [8]. It is possible that currently available assays cannot detect all potential aPL. Moreover, new specificities are tested in only a few research laboratories. Some patients that are “seronegative” for aPL may occasionally have antibodies against other membrane phospholipids such as phosphatidylserine, phosphatidic acid and phosphatidylinositol, that are not determined in routine blood test [14]. In this respect, phosphatidylethanolamine antibodies (aPE) are detected in stroke patients with suspected aPL in the absence of LA or aCL antibodies [15]. 

We cannot exclude that CVT in our patients might be due to other aPL factors, such as aPE. Unfortunately, these antibodies are not routinely assessed in our laboratory. On the other hand, our report suggests that, in patients with CVT, primary SS should be suspected, even in the absence of thrombophilic risk factors. 

In a large series of Italian SS patients, 5.8% of them had CNS involvement. None of them presented with CVT. However, 50% presented with “subacute encephalopathy” characterized by memory loss, cognitive dysfunction, and visual disturbances [16]. Whether some of these cases might be due to unrecognized CVT remains undetermined. 

In conclusion, CNS impairment in SS is probably more heterogeneous than commonly believed. A well-defined management protocol awaits studies with larger case numbers [16] while specific criteria and guidelines for primary CNS SS are still lacking. 

In patients with CVT primary SS should be suspected, even in the absence of thrombophilic risk factors. Since conventional laboratory testing may fail to detect uncommon coagulopathies, it might be wise to repeat the screening tests in more than one occasion. When possible, patients should be screened for other and more rare aPL. 

## Figures and Tables

**Table 1:** Summary of clinical, biological, and radiologic findings in the 2 patients.

Cases	Sicca syndrome	Anti-SSa/SSb	Chisolm's score	Vitali criteria
Patient 1	Present	Present	4	4/6
Patient 2	Present	Present	>5	4/6
